# Saliva Proteome, Metabolome and Microbiome Signatures for Detection of Alzheimer’s Disease

**DOI:** 10.3390/metabo14120714

**Published:** 2024-12-19

**Authors:** Maxime François, Dana Pascovici, Yanan Wang, Toan Vu, Jian-Wei Liu, David Beale, Maryam Hor, Jane Hecker, Jeff Faunt, John Maddison, Sally Johns, Wayne Leifert

**Affiliations:** 1 Nutrition and Health Program, Molecular Diagnostic Solutions Group, CSIRO Health & Biosecurity, Adelaide, SA 5000, Australia; 2 CSIRO Health & Biosecurity, Westmead, NSW 2145, Australia; 3 CSIRO Health & Biosecurity, Microbiomes for One Systems Health-Future Science Platform, Adelaide, SA 5000, Australia; 4 CSIRO Environment, Agricultural and Environmental Sciences Precinct, Acton, Canberra, ACT 2601, Australia; 5 Metabolomics Unit, CSIRO Environment, Ecosciences Precinct, Dutton Park, QLD 4001, Australia; 6 Department of Internal Medicine, Royal Adelaide Hospital, Adelaide, SA 5000, Australia; 7 Department of General Medicine, Royal Adelaide Hospital, Adelaide, SA 5000, Australia; 8 Aged Care Rehabilitation & Palliative Care, SA Health, Modbury Hospital, Modbury, SA 5092, Australia

**Keywords:** Alzheimer’s disease, metabolomics, proteomics, saliva, microbiome, systems biology

## Abstract

**Background:** As the burden of Alzheimer’s disease (AD) escalates with an ageing population, the demand for early and accessible diagnostic methods becomes increasingly urgent. Saliva, with its non-invasive and cost-effective nature, presents a promising alternative to cerebrospinal fluid and plasma for biomarker discovery. **Methods**: In this study, we conducted a comprehensive multi-omics analysis of saliva samples (*n* = 20 mild cognitive impairment (MCI), *n* = 20 Alzheimer’s disease and age- and *n* = 40 gender-matched cognitively normal individuals), from the South Australian Neurodegenerative Disease (SAND) cohort, integrating proteomics, metabolomics, and microbiome data with plasma measurements, including pTau181. **Results**: Among the most promising findings, the protein Stratifin emerged as a top candidate, showing a strong negative correlation with plasma pTau181 (r = −0.49, *p* < 0.001) and achieving an AUC of 0.95 in distinguishing AD and MCI combined from controls. In the metabolomics analysis, 3-chlorotyrosine and L-tyrosine exhibited high correlations with disease severity progression, with AUCs of 0.93 and 0.96, respectively. Pathway analysis revealed significant alterations in vitamin B12 metabolism, with Transcobalamin-1 levels decreasing in saliva as AD progressed despite an increase in serum vitamin B12 levels (*p* = 0.008). Microbiome analysis identified shifts in bacterial composition, with a microbiome cluster containing species such as *Lautropia mirabilis* showing a significant decrease in abundance in MCI and AD samples. The overall findings were reinforced by weighted correlation network analysis, which identified key hubs and enriched pathways associated with AD. **Conclusions**: Collectively, these data highlight the potential of saliva as a powerful medium for early AD diagnosis, offering a practical solution for large-scale screening and monitoring.

## 1. Introduction

Alzheimer’s disease (AD) stands as the primary neurodegenerative form of dementia, marked by cognitive decline, memory loss, and behavioural changes [1,2]. With a global ageing population, AD incidence is projected to surge, particularly in developing nations, raising substantial societal and economic concerns [3,4]. Early disease stages, often manifested as mild cognitive impairment (MCI), present a crucial intervention window to modify risk factors and mitigate cognitive decline. Alarmingly, a significant proportion of individuals with MCI progress to AD annually, necessitating the identification of those at heightened risk for timely preventative measures [5]. However, early AD detection poses a formidable challenge, underscoring the urgent need for innovative and reliable biomarkers to enhance diagnostic accuracy.

Present diagnostic approaches for AD are insufficient, leaving patients in prolonged states of suffering due to their invasive, costly nature and limited biomarker identification capabilities. To enable successful population-based AD screening, accessible, minimally invasive, and cost-effective diagnostic methods with high specificity and sensitivity are imperative. While previous research utilised buccal mucosa cells [6,7,8,9,10] as a minimally invasive approach to identify potential AD biomarkers, saliva has gained prominence as a body fluid for biomarker discovery. A minimally invasive strategy utilising peripheral blood biomarkers such as phosphorylated-tau levels [11,12] shows promise for early AD diagnosis, enabling the implementation of preventative strategies to mitigate clinical symptoms. Nonetheless, the current ability to detect early AD stages and their progression remains limited, necessitating a minimally invasive approach to identify preclinical individuals with AD and MCI.

Despite numerous hypotheses, the precise mechanisms underlying AD onset and progression remain elusive. Recent advances in proteomics and metabolomics have offered valuable insights into the molecular underpinnings of AD. Recent developments in saliva proteomics have facilitated the identification of saliva proteins, with approximately 40% of blood proteins used in diagnostics also present in saliva [13]. Targeted metabolomics in blood and saliva have unveiled novel AD biomarkers, while integrated metabolomic and proteomic analyses of saliva have elucidated impacted metabolic pathways contributing to disease presentation [14,15]. Additionally, the composition of bacteria in the saliva shifts according to the stage of cognitive decline, with a preference towards opportunistic species over periodontal bacteria [16], and those with AD exhibited a significantly lower variety and richness in their saliva’s microbial content compared to healthy individuals, whilst changes were also seen in those with the APOEε4 allele [17].

This study explores the proteomic and metabolomic signatures, including the microbiome of saliva, in cognitively normal individuals and those with MCI and AD and links them with several plasma measures of the same patients, most importantly their plasma phosphorylated Tau181 levels. We identify several specific protein and metabolite biomarkers with similar or better classification performance as plasma pTau181 in this cohort, thus seeking to enhance early detection methods that could be readily self-administered. Additionally, comparing the metabolomic and proteomic profiles of saliva from patients with different levels of cognitive impairment highlights that certain cellular pathways may change as the disease progresses (e.g., from mild cognitive impairment to Alzheimer’s disease), providing insights into Alzheimer’s pathology and potential therapeutic targets.

The microbiome analysis available for a subset of the cohort further strengthens the results. Taken together, the three omics confirm enriched relevant pathways, including those associated with AD and vitamin B12 metabolism, and demonstrate the usefulness of saliva as a window into systems-level changes associated with disease progression.

## 2. Materials and Methods

### 2.1. Patient Samples

This cross-sectional study received approval from the relevant Human Research Ethics Committees, including CSIRO HREC (09/11). All procedures followed were in strict accordance with the committees’ guidelines, and every participant gave written informed consent before their inclusion in the study. The identification of Mild Cognitive Impairment (MCI) or Alzheimer’s Disease (AD) in participants was carried out by gerontology clinicians (Hecker, Faunt, Johns and Maddison) following the criteria set by the National Institute of Neurological and Communicative Disorders and the Stroke-Alzheimer’s Disease and Related Disorders Association (NINCDS-AD&DA) [18] and incorporating recommendations from the National Institute on Aging-Alzheimer’s Association (NI-AAA) workgroups for diagnosing MCI using clinical criteria [19]. The determination of dementia due to AD was made in a clinical context, aligning with the “probable AD” criteria outlined in the NI-AAA guidelines [20]. Mini-Mental State Examination (MMSE) was assessed to verify cognitive impairment.

The data presented in this study encompass a total of 80 participants from the South Australian Neurodegenerative Disease (SAND) cohort, which includes (1) a cognitively normal (CN) group of 40 healthy participants (21M/19F) matched by age and gender (as summarised in Supplementary Table S5), (2) 20 individuals clinically diagnosed with MCI (9M/11F); and (3) 20 individuals clinically diagnosed with AD (12M/8F), 65% of AD patients received acetylcholinesterase inhibitor therapy. The study excluded patients with significant cognitive impairments due to head trauma, alcoholism, learning disabilities, or Parkinson’s disease. Additionally, exclusion criteria for all participants included those undergoing cancer treatment with chemotherapy or radiotherapy and those consuming micronutrient supplements in dosages above the recommended intake.

### 2.2. Sample Collection, Biochemical Measurements

Non-fasting blood and saliva were collected and processed within 3 h of the collection as described previously [14,15], typically between 11 a.m. and 2 p.m. No food was consumed up to 2 h prior to collection. However, drinking water was encouraged. For the collection of saliva, we employed the use of RNAPro•SAL™ kit (Oasis Diagnostics^®^ Corporation, Vancouver, WA, USA), as per the manufacturer’s instructions (non-stimulated). All samples were stored at –80 °C until analysis. Plasma vitamins were measured by the commercial clinical laboratory service SA Pathology (Adelaide, South Australia, Australia).

### 2.3. Apolipoprotein E Genotyping

APOE genotyping for alleles ε2, ε3 and ε4 was based on allele-specific PCR methodology adapted to real-time PCR monitored by TaqMan probe as described previously [21].

### 2.4. Chemicals

All chemicals used were Mass spectrometry grade or higher and purchased from Sigma-Aldrich (Castle Hill, NSW, Australia) unless specified otherwise. Bradford assay reagents were purchased from BioRad Laboratories (Gladesville, NSW, Australia). The Proteomics FlexMix Calibration Solution and Retention Time Calibration Mixture for liquid chromatography (LC) were acquired from Thermo Scientific (Woolloongabba, QLD, Australia). The gas chromatography (GC) retention time calibration standard and isotopically labelled standards were sourced from Cambridge Isotope Laboratories (Tewksbury, MA, USA). Phosphate buffered saline (PBS) and Polyethersulfone (PES) filters were from GibcoTM, Life Technologies Corporation (Parkville, VIC, Australia).

### 2.5. Untargeted Metabolomics

Metabolomics was conducted on saliva samples as described earlier [15]. Briefly, saliva samples were processed with internal standards before being derivatised for GC-MS analysis. The analysis was conducted on an Agilent GC-MS system (Agilent Technologies, Mulgrave, VIC, Australia), and data were processed using MassHunter and Mass Profiler Professional software (v14.9, Agilent Technologies, Mulgrave, VIC, Australia). Quality control was ensured with a sequence of 80 samples, achieving residual standard deviations of 6.8%, 4.1%, and 4.9% for the internal standards.

### 2.6. Untargeted Proteomics

Saliva proteomics was conducted on saliva samples as described earlier [15]. Briefly, proteins were precipitated with cold acetone, air-dried, and then redissolved in 8M urea. Protein concentration was determined using a Bio-Rad Bradford assay kit, and a 5 µg sample was reduced and alkylated before trypsin digestion at 37 °C overnight. Digestion was stopped with 1% formic acid, and the solution was filtered through a 0.22 µm filter. Tryptic peptides (100 ng) were purified and concentrated using a PepMap100 C18 trap column, then separated on a nanoscale PepMap100 C18 column using a Thermo Scientific Ultimate™ 3000 RSLC nano LC system. Mobile phase A was water with 0.1% formic acid, and mobile phase B was 80% acetonitrile with 0.08% formic acid. A gradient elution was applied from 5% to 40% solvent B over 60 min, followed by a rapid increase to 99% in 10 min. Peptides were ionised using a Nanospray Flex Ion Source (Thermo Scientific, Waltham, MA, USA) at 2.3 kV and 300 °C. Mass spectrometry, including MS1 and MS/MS, was performed using an Orbitrap Fusion MS. The peptidic sample loaded was 167 ng. MS scans ranged from 400 to 1500 *m*/*z* with a 120K resolution at 200 *m*/*z*, an AGC target of 4 × 10^5^, and a maximum injection time of 50 ms. MS/MS targeted abundant precursors (2+ to 7+ charge states, intensities above 1 × 10^5^) with a 1.6 *m*/*z* quadrupole isolation window, 28% HCD collision energy, and rapid scan rate detection in the ion trap. MS/MS settings included an AGC target of 4 × 10^3^, a maximum injection time of 300 ms, and a 15 s dynamic exclusion, operating in top speed mode for three-second MS and MS/MS scan cycles.

Peptide and protein analysis and quantification were conducted using Protein Discoverer 2.2 and the Sequest HT search engine. Spectral data were matched to the UniProt Homo sapiens database (Proteome ID: UP000005640) with a precursor mass tolerance of 10 ppm and product ions analysed at 0.6 Da, allowing up to three missed tryptic cleavages. Modifications included Oxidation (+15.995 Da), Deamidation (+0.984 Da), Amidation (−0.984 Da) and Propionamidation (+71.037 Da). Peptide matches were validated with the Percolator algorithm, maintaining a 1% FDR using q-values. Relative abundance was calculated from normalised precursor intensity, with ratios derived from summed abundance, and statistical significance assessed by ANOVA (R version 4.4 base packages).

### 2.7. Saliva Microbiome

#### 2.7.1. Saliva Sample Processing and DNA Extraction

All subset saliva samples (*n* = 51, comprising 22 controls, 13 MCI and 16 AD; full sub-cohort characteristics are included in Supplementary Table S5) were aliquoted at 100 µL each and kept frozen at −80 °C prior to DNA extraction. Saliva samples were thawed at room temperature for 5 min and then homogenised with 100 µL 1X PBS.

QIAamp^®^ DNA Mini Kit (Qiagen) was used to extract DNA. The saliva samples were suspended in 180 µL Lysis Buffer ATL and 20 µL of Proteinase K provided in the kit, then mixed by briefly vortexing. The mixtures were incubated at 56 °C for 2 h. The DNA extraction process followed the Quick-Start Protocol provided on the Qiagen website (https://www.qiagen.com/au/resources/resourcedetail?id=566f1cb1-4ffe-4225-a6de-6bd3261dc920&lang=en) (accessed on 1 September 2023). After all purification and washing steps, the QIAamp^®^ Mini spin columns (Qiagen; Clayton, VIC, Australia) containing purified DNA were placed in 1.5 mL microcentrifuge tubes, then added with 50 µL Elution Buffer AE and incubated at room temperature for 1 min. The purified DNA was then eluted from the spin column by centrifugation at 6000× *g* and kept at −20 °C for downstream analysis.

#### 2.7.2. DNA Sequencing and Analysis

Amplicon sequencing of bacterial full-length 16S rRNA genes was performed using the PacBio HiFi sequencing platform (Australian Genome Research Facility, Brisbane, Australia). Sequence reads were analysed with the QIIME2 pipeline. Saliva samples of 2 healthy individual donors were used as a positive control for the DNA extraction process.

### 2.8. Statistical Analysis

The extracted mass spectrometry abundance data from saliva proteins and metabolites, as well as the microbiome abundance data, was analysed using a consistent workflow. Data was log-transformed and median normalised, and missing values were imputed by sampling from a Gaussian distribution downshifted by 1.8 standard deviations, with a width of 0.3 (Perseus platform standards [22]). Differential expression between all categories considered (control–AD, control–MCI and MCI–AD) was carried out using moderated *t*-tests as implemented in the limma package [23]; multiple testing corrected values were also generated using the Benjamini and Hochberg fdr-correction. Spearman rank correlation values and corresponding *p*-values were generated for all analytes and corresponding plasma measurements: pTau181, age—using samples stratified by control and case (MCI and AD combined)—homocysteine and Vitamin B12.

For protein subsets of interest, pathway enrichment was carried out using Cytoscape version 3.10.2 [24], and for metabolites using MetaboAnalyst version 6.0 [25]; metabolite disease associations were downloaded from the Human Metabolite Database [26].

### 2.9. Weighted Correlation Network Analysis

The WGCNA R package version 1.72 [27] was used to cluster all saliva omics data separately, using the specific workflow detailed in [28]. Normalised, imputed and log transformed data were used as inputs, with default parameters of Rcutoff 0.85, minimum module size 20, soft threshold generated and average topology overlap measure (TOM) distance. Module eigenfeatures were outputted and plotted with respect to disease status, and the top hub proteins were listed. Module trait correlations of the module eigenfeatures with all the clinical and phenotypic covariates available were generated and plotted as a labelled heatmap. Modules with a significant association with the MMSE score (correlation *p*-value < 0.001) were highlighted.

### 2.10. Biomarker Selection

For each of the omics datasets, markers were ranked based on their ability to discriminate between Control and AD, Control and MCI, and MCI and AD. Fold change and unpaired *t*-test *p*-values were generated using the limma R package, area under the curve (AUC) was calculated for each marker using the *roc* function from the pROC R package [29], with the marker abundance as the continuous variable and the disease classification as the binary variable.

In addition, a more complete variable selection workflow was followed to rank markers that can distinguish between control and MCI + AD more robustly based on importance scores using several methods: LASSO regression (glmnet R package implementation [30]), extreme gradient boosting (xgboost R package), and gradient boosting (gbm R package), on random subsamples of the data, with the selection repeated 100 times. For LASSO and xgboost, five-sixths of the data were subsampled at random, and variables were ranked in decreasing order of importance. For gbm, a training fraction of 75% was used each time. The resulting top 20 markers ranked by importance for each of the three methods were then combined and summed up to generate a single overall rank, aiming to capture markers that are both stable under random subsample selection and under different methodologies used for variable selection.

When considering panels of analytes (proteins or metabolites), classification potential was determined using a logistic model, with disease category (control/case) as the outcome, and all features considered as predictors; leave-one-out cross-validation (LOOCV) was used to assess the performance of each model, where data were trained, in turn, on all but one sample, and the prediction was made on the remaining sample. Wherever analyte panels were considered, LOOCV probabilities were used as the continuous variable to assess the AUC to avoid over-fitting.

## 3. Results

### 3.1. Plasma Protein Profiles Changes with Disease Progression and Correlations with Saliva Proteins

**Figure 1:**
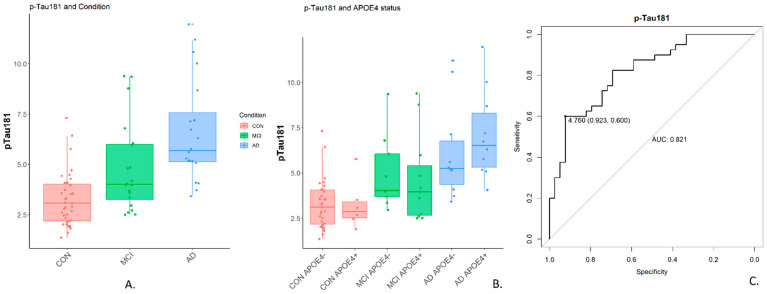
The levels of pTau181. (**A**): pTau181 (pg/mL) in plasma measured by SIMOA by condition; (**B**): pTau181 in plasma by condition and APOEε4 status; (**C**): pTau181 ROC curve (Control vs MCI and AD) with area under the curve and threshold of maximum sensitivity + specificity. pTau181 levels differ significantly between control and AD (*p*-value < 0.001), control and MCI (*p*-value 0.01) and MCI and AD (*p*-value 0.01). Full two-way ANOVA summaries and post hoc Tukey analysis results are included in the Supplementary Information.

In addition to the blood measurements previously described for this cohort [15], additional measurements were undertaken and are summarised in Table 1, alongside age, sex and MMSE score. The levels of pTau181 (Figure 1, *p* < 0.001) and serum Vitamin B12 (*p* = 0.008) showed a significant increase in plasma with disease severity in this cohort (Table 1).

Given the significant predictive capacity of pTau181 observed in this study, the next step was to identify saliva biomarkers with a high and significant correlation (positive or negative) with the plasma pTau181 levels. These biomarkers could offer a non-invasive saliva-based method for indicating increased pTau181 plasma levels.

The proteomics, metabolomics and microbiome analysis yielded 751 proteins, 174 metabolites and, respectively, 115 microbiome species. Regarding completeness of detection, prior to data imputation, 61% of the proteins were quantitated in 50% or more of the samples; all metabolites were quantitated in more than 50% of the samples, and respectively, 52% of the microbiome species were quantitated in more than 50% of the 51 samples present in the microbiome analysis. The number of quantitation values present prior to imputation in each of the categories (AD, MCI and control) is listed in Supplementary Tables S7, S9 and S10 for each individual analyte present in each of the three omics analyses.

Supplementary Tables S1 and S2 provide correlations of all measured saliva proteins and metabolites, as well as correlations with other plasma measurements, including age, homocysteine and Vitamin B12.

Table 2 shows several saliva proteins had moderate negative correlations with plasma pTau181 levels (Pearson correlation < −0.4, *p* < 0.001), including Stratifin (14-3-3 protein sigma, with the highest negative correlation of −0.49), Cornifin-A (Small proline-rich protein 1), Small proline-rich protein 3 (known to co-express with Cornifin-A), Aldehyde dehydrogenase, dimeric NADP-preferring, Heme-binding protein 2 and 14-3-3 protein eta (known to co-express and interact with Stratifin). Of the top 10 most highly correlated proteins to pTau, all were down-regulated with increased disease severity and have negative correlations to pTau181. Among these 10 proteins, five (14-3-3 protein sigma and eta, and the three small proline-rich proteins) are keratinocyte proteins, and the top enriched biological process for this small subset is keratinisation. Of these 10 proteins, small proline-rich protein 2D exhibits a high positive correlation with age (control samples only—Supplementary Table S1).

Similarly, Table 2 presents the top 20 metabolites ranked by their correlation with plasma pTau181. Unlike in the proteomics data, the metabolites show a mix of both positive and negative correlations. The highest positive correlations are with 3-Chlorotyrosine, 2-amino-2-methyl-1,3-propanediol, L-tyrosine, hydroxyphenyllactic acid and citramalic acid. The lowest negative correlations were with beta-gentiobiose, maltose, maltotriose, D-malic acid, urea and cellotetraose.

### 3.2. Saliva Protein and Metabolite Clusters Show Patterns That Correlate with Disease Progression

The subsequent analysis concentrated on global, systems-level changes that could be observed in the saliva proteome and metabolome, and for this, we employed a correlation network approach. Weighted gene correlation network analysis (WGCNA) was developed originally for the study of gene networks [31] and has also been shown to be a powerful tool for the study of protein correlation networks [28], including in the specific context of neurodegeneration [32,33]. We applied it here to generate clusters of tightly correlated proteins/metabolites called modules and to further select those showing significant associations with disease progression (the MMSE score serves here as the proxy for disease severity increase) and with the available plasma biomarker measurements. This was accomplished by determining the module eigenfeature (which may be referred to as eigengene, eigenprotein and so on, depending on the relevant omics to which it is employed), defined as a principal component of the respective module data and can be thought of as a weighted average expression profile for the cluster. The eigenfeature can then be used for two goals: firstly, to correlate against all available phenotypic and blood plasma information related to the samples—referred to as “module–trait correlations”. Secondly, markers in a module can be ranked by their correlation with the module eigenfeature (kME value), which then provides a ranking of the members of the module, with those at the top more likely to be highly connected “hub” proteins/metabolites and thus candidate important biological markers.

**Figure 2:**
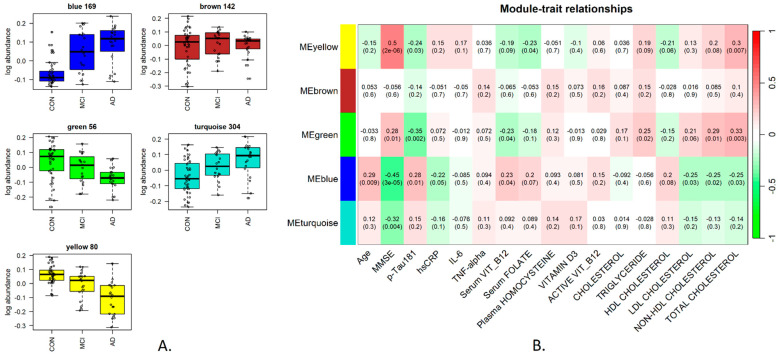
Saliva proteins network correlation analysis: five clusters of proteins were identified: blue (169 proteins), brown (142 proteins), green (56 proteins), turquoise (304 proteins) and yellow (80 proteins). Panel (**A**) shows the pattern of the module eigenprotein across the disease course. Panel (**B**) shows the correlation of each module eigenprotein (ME) with other traits, namely all phenotypic variables and plasma measurements in the study. The values are shown in the format correlation (*p*-value) for each respective module/variable pair.

For the saliva proteomics, five clusters were obtained (Figure 2A), of which two significantly correlated with the MMSE score as shown in Figure 2B: the yellow cluster contained 80 proteins and had the highest positive correlation with the MMSE score (0.5, *p* < 0.001); the blue cluster contained 169 proteins and showed the largest negative correlation with the MMSE score (−0.45, *p* < 0.001). Both clusters also showed representative trend correlation with plasma pTau181, albeit at slightly lower significance levels. The cluster membership and kME values for all saliva proteins are available in Supplementary Table S3.

Pathway analysis was carried out using Cytoscape [24] for all saliva protein clusters and showed Vitamin B12 metabolism (including transcobalamin 1, Vitamin B12 transporter) and folate metabolism enriched in the yellow cluster, which showed a decreasing trend with disease progression. Transcobalamin-1 was also one of the top 5 hub proteins in the yellow cluster, suggesting an important systems-level role (Table 3).

In the case of the blue cluster, which exhibited an increasing module trend with disease progression, several enriched pathways were identified, including those related to amyotrophic lateral sclerosis and Alzheimer’s disease (Table 3). Most proteins identified from the AD pathway were alpha and beta tubulins, whereas the hub proteins were predominantly nuclear Histone 2A and Histone 2B proteins.

**Figure 3:**
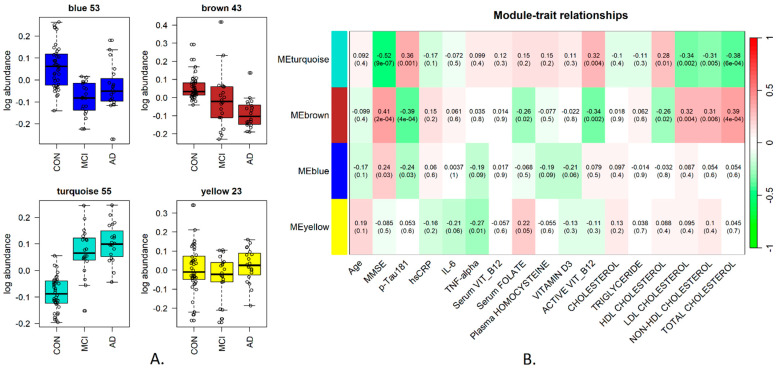
Saliva metabolite network correlation analysis: five clusters of metabolites were identified: blue (53 metabolites), brown (43 metabolites), turquoise (55 metabolites) and yellow (23 metabolites). Panel (**A**) shows the pattern of the module eigenmetabolite (ME) across the disease course. Panel (**B**) shows the correlation of each module eigenmetabolite with other traits, namely all phenotypic variables and plasma measurements in the study. The values are shown in the format correlation (*p*-value) for each respective module/variable pair.

For the saliva metabolomics, four modules were obtained (Figure 3A), of which two significantly correlated with the MMSE score (Figure 3B); the brown cluster contained 43 metabolites and showed the highest positive correlation with MMSE (0.41, *p* < 0.001). The turquoise cluster contained 55 metabolites and showed the lowest negative correlation with MMSE (−0.52, *p* < 0.001, Figure 3B). Both clusters also showed significant correlations with plasma pTau181 in opposite trends to the MMSE and significant positive correlations with total cholesterol (Figure 3B). The cluster membership and kME values for all saliva metabolites are available in Supplementary Table S4.

Pathway analysis was carried out using MetaboAnalyst [25] and showed alanine, aspartate and glutamate metabolism, valine, leucine and isoleucine biosynthesis and arginine biosynthesis enriched in the turquoise cluster, which showed a module trend increasing with disease progression. A MetaboAnalyst metabolite-disease interaction network analysis of the turquoise cluster highlighted Schizophrenia and AD as the top two diseases in terms of degree connectivity, with 11 metabolites in the cluster having an association with AD in the Human Metabolite Database (HMDB)—Supplementary Figure S1.

Interestingly, the top hub metabolites in the turquoise module include 3-chlorotyrosine and 2-amino-2-methyl-1,3-propanediol, the two metabolites showing the highest individual correlations with plasma pTau181 (Table 2). Similarly, the top hub metabolites in the brown module include beta-gentiobiose, maltose, maltotriose and cellotetraose (Table 3), which all have high negative individual correlations with plasma pTau181. Thus, the two statistical approaches reinforce each other and likely capture biologically relevant information.

### 3.3. Saliva Microbiome Clusters

The same correlation network approach was run to obtain clusters for the microbiome saliva data available for this cohort (*n* = 51 samples based on sample availability—Supplementary Table S5 shows the characteristics of this subset of the cohort). Three distinct clusters were obtained, with the largest (turquoise cluster containing 88 species) showing an overall decrease in abundance in MCI and AD samples (Supplementary Figure S2). Supplementary Tables S6 and S7 show both the cluster information and the overall differential expression statistics. Interestingly, the turquoise cluster eigenfeature shows a significant correlation with Vitamin B12 metabolism, and three of the top 10 hub species in the cluster belong to the *Lautropia* genus (*Lautropia dentalis*, *Lautropia mirabilis*). Similarly, the blue cluster contains 24 species and an eigenfeature showing an increase in disease. The hub species include two *Veillonella atypica* and four *Prevotella* species (Supplementary Table S6).

### 3.4. Saliva Proteins and Metabolites—Biomarkers

**Figure 4:**
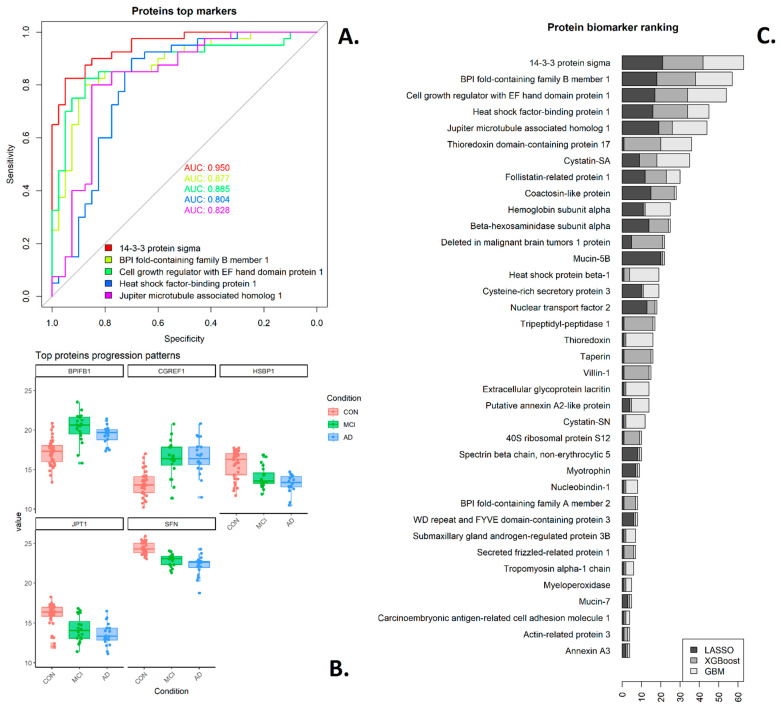
Saliva protein biomarker ranking. (**A**) ROC and AUC for top five-ranked biomarkers. (**B**) Boxplots of protein abundance for top five biomarkers. (**C**) The combined rankings from three variable selection methods where the ranking was based on the number of times the respective protein was selected amongst the top 10 most important markers by each method.

**Figure 5:**
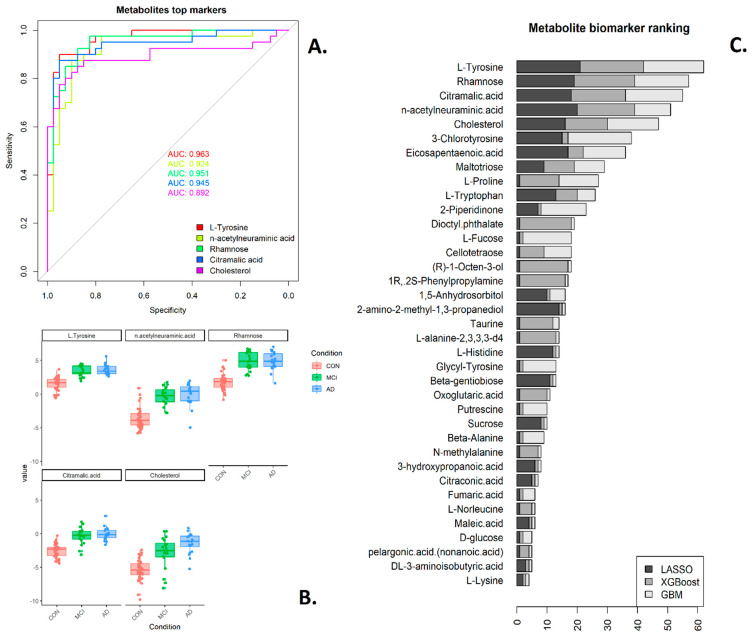
Saliva metabolite biomarker ranking. (**A**) ROC and AUC for top five ranked biomarkers. (**B**) Boxplots of protein abundance for top five ranked biomarkers. (**C**) The combined rankings from three variable selection methods where the ranking was based on the number of times the respective metabolite was selected amongst the top 10 most important markers by each method.

While the pathway and correlation network analyses canvass data at a systems level, biomarker discovery workflows aim to find a small subset of markers (proteins, metabolites, etc.) which can classify disease categories or disease progression with high accuracy and, as such, could be used in a diagnostic or monitoring test. Such workflows fall into two broad categories [34], either starting with a univariate ranking of each marker in terms of fold change, *p*-value or area under the curve or with multivariate selection methods that rank variables by importance, such as LASSO, boosted gradient methods, and random forests, amongst many others. The latter approaches are usually run multiple times on data sub-selections, with performance assessed using cross-validation to increase robustness. For classification problems with more than one class, one can undertake pairwise comparisons or a multi-class approach [35]; for this study, we have undertaken a combination. We initially aimed to find saliva markers that distinguish between control (*n* = 40) and disease (MCI and AD combined, *n* = 40), firstly as a matter of practicality given the dataset size, then compared the categories of MCI and AD separately (20 vs. 20 samples) to find saliva markers that can detect disease progression. For the combined comparison, AUC values are reported as “one vs. all”; thus, control vs. MCI and AD combined [36]. Differential expression results and AUC values for all the separate individual comparisons (MCI–control, AD–control, MCI–AD) were also carried out, and the results are recorded in Supplementary Tables S9 and S10; it is expected to have substantial overlap between the approaches, specifically markers that separate well control from MCI and AD combined should also distinguish control from AD and control from MCI. Rankings obtained from three variable selection methodologies (LASSO, XGBoost and GBM) were combined, resulting in a ranked list of top proteins and metabolites possible biomarkers, included as a bar plot in Figure 4 (saliva proteome) and Figure 5 (saliva metabolites). Supplementary Table S8 also summarises the classification potential using the whole set (*n* = 80), showing each feature’s area under the receiver operator curve (AUC).

In the case of proteins (Figure 4), the top-ranked marker was Stratifin (14-3-3 sigma), which independently had the highest area under the ROC curve (AUC~0.95) and was also the saliva protein with the highest correlation with the plasma pTau181 protein (cor –0.48, *p*-value < 0.001).

For metabolites (Figure 5), the top-ranked markers included L-tyrosine (AUC 0.96), rhamnose (AUC 0.95), citramalic acid (AUC 0.94), n-acetylneuraminic acid (0.92), cholesterol (AUC 0.89) and 3-chlorotyrosine (AUC 0.93). Of those, L-tyrosine, 3-chlorotyrosine and citralmic acid also had high individual correlations with plasma pTau181, and 3-chlorotyrosine and rhamnose were amongst the top hub metabolites in the turquoise module based on correlation network analysis. Therefore, the statistical-based approaches overlap both the systems-level and biological methodologies. Saliva 3-chlorotyrosine thus appears to be a key metabolomics marker via all orthogonal approaches undertaken here; it is an oxidative product, a marker of oxidative damage in proteins, which is itself an early marker of AD [37]. A search of the top five ranking metabolites against the Human Metabolite database [26] for metabolite and disease showed that L-tyrosine was found to be altered in Alzheimer’s disease in a variety of studies and biospecimens, including blood, CSF, urine and saliva [38,39].

For the progression comparison (20 MCI vs. 20 AD cases), the leading candidate was Transcobalamin-1 (AUC 0.83, decreasing in AD samples—Supplementary Figure S3), which interestingly was also identified via the WGCNA analysis as a hub protein in the yellow module of saliva proteins downregulated with disease, as well as being involved in Vitamin B12 metabolism, alongside SAA1 and SERPINA3 (Table 3).

### 3.5. Protein Panels

**Figure 6:**
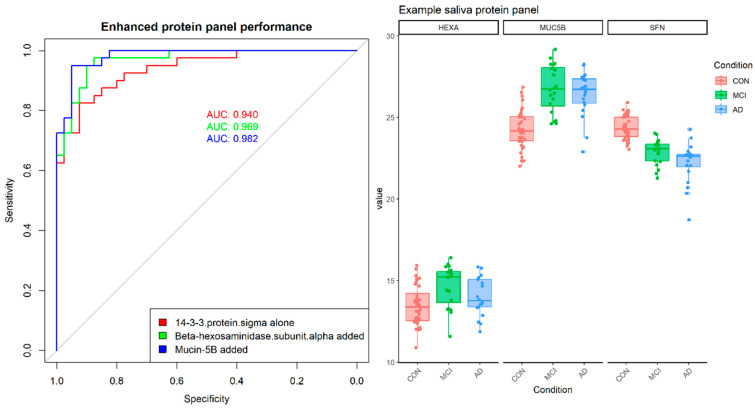
Optimal protein panel. The three-protein panel was selected by starting with Stratifin (14-3-3 sigma) and incrementally adding proteins that provided the largest increase in performance as measured by the area under the curve. The resulting AUC is 0.98 (logistic regression, leave one out cross-validation).

The classification performance of single biomarkers is already strong in this cohort but can be further improved by identifying combinations of markers; given the size of the datasets, we aimed for small (*n* < 5) panels, as small panels are more robust, less prone to over-fitting and have a more realistic chance to capture the complexity of body fluid proteomics [40]. Here, the classification potential of a panel of markers was assessed using a logistic model, and the performance was measured by the AUC, using a leave-one-out cross-validation to minimise over-fitting (Figure 6). The starting point was the best-performing marker (in the case of proteins, Stratifin, in the case of metabolites L-tyrosine), and more markers were added iteratively from amongst the set of potential biomarkers (*n* = 37, Figure 4) at each stage, adding the marker that would lead to the biggest improvement in performance as measured by AUC.

In the case of Stratifin (14-3-3 sigma), the resulting optimal panel included Stratifin, Mucin-5B and Beta-hexosaminidase subunit alpha (AUC = 0.982), achieved a sensitivity of 0.95 at 0.95 specificities using a logistic model with leave one out cross-validation.

In the case of metabolites, there are multiple markers with stand-alone AUC > 0.90 and two markers with AUC > 0.95 (L-tyrosine, AUC = 0.96 which is also amongst the top most correlated metabolites with plasma pTau181 levels, and rhamnose, AUC = 0.95); their performance via logistic models could not be improved by adding more markers, though several other panels of similar performance could be identified from amongst the top-ranked metabolite markers.

## 4. Discussion

Plasma pTau has been shown to correlate with CSF pTau and to be a useful diagnostic and prognostic biomarker for MCI and AD in various clinical studies with good classification performance (various AUC ranges reported: 0.84–0.94 [41], 0.83 [42]); this current study recapitulates those findings, with current study plasma pTau181 AUC 0.82. Though plasma is much less invasive than CSF, saliva is much more readily accessible and thus amenable to self-monitoring. Whilst pTau was not detected in saliva via mass-spectrometry (though it can be detected via Lumipulse enzymatic light emitting assay [43]), this study highlighted several saliva markers that show good correlation with plasma pTau181 and indeed found that some of the closest correlates (Stratifin, L-tyrosine, rhamnose) provided better classification performance on this current cohort (AUC’s 0.95). The top protein correlate of pTau181, Stratifin (14-3-3 sigma), is involved in the biological process of keratinisation, as are, in fact, three others of the top 10 protein correlates, all small proline-rich proteins. This is intriguing due to the known association of Alzheimer’s disease with the “unfolded protein response” signalling pathway and the impact it has on keratinocytes [44].

In addition to exploring correlations with the available plasma pTau181 concentrations, this study considered two additional orthogonal approaches: using a systems-level network correlation analysis of the saliva proteomics and metabolomics data and carrying out several independent machine learning approaches to rank promising saliva biomarkers. The network analysis identified clusters of proteins that correlated with disease progression and highly connected hub proteins likely to be of interest within them. The biomarker ranking workflow sought features that separate the conditions well under different computational methods and different sub-samples of the cohort. Then, we focused on re-enforcing signals common to several analyses to propose saliva markers with predictive power and likely biological relevance. This approach complements the pathway-level analysis of this cohort carried out in [15] and the ELISA-based proof of concept, which focused on a small set of inflammatory markers [45]; the latter subset included Stratifin and Cystatin and, as such, provides an orthogonal method validation of these potential biomarkers.

The top protein biomarker Stratifin (14-3-3 protein sigma, and additionally noting 14-3-3 proteins delta and zeta) was previously identified as downregulated in a comparison of pooled saliva specimens from patients with breast cancer (stage 2 invasive ductal carcinoma of the breast, IDC) compared to controls [46]; Stratifin has a broad involvement in various diseases and in general the 14-3-3 protein family plays a role in DNA damage repair, inflammation and apoptosis [47]. Interestingly, we found increased DNA damage in buccal cells (and lymphocytes) from individuals with AD in our previous studies [6,10,48].

Some common threads emerged from the systems-level observations across proteins, metabolites and microbiomes. Notably, the relevance of vitamin B12 metabolism, seen in our data to decrease in saliva with disease progression, though levels of serum vitamin B12 in plasma increased with the disease (note a similar observation measuring various serum and saliva proteins including transcobalamin-1 in matched samples show negative, not significant correlations [49]). Transcobalamin-1 was also the top-ranked biomarker for the progression comparison of MCI versus AD samples (AUC 0.83, Supplementary Figure S3), which showed decreased levels in AD saliva compared to MCI saliva in this study. Transcobalamin is one of the three soluble binding proteins involved in the transport and uptake of vitamin B12 (cobalamin); specifically, transcobalamin binds to vitamin B12 with high affinity [50] and the resulting complex is recognised by receptors, which then facilitate the uptake. The possible links between vitamin B12 deficiency and cognitive deficits, including dementia and AD, have been extensively explored and reviewed in the literature [51,52]; thus, the current saliva study adds an intriguing point of interest.

This pilot study utilised 16S rRNA sequencing to characterise the oral microbiome of individuals with normal cognition and those with MCI and AD. We hypothesised that the oral microbiome would be linked to Alzheimer’s disease and proteomics and metabolomic biomarkers. Our results supported previous findings, where *Lautropia mirabilis* was identified as one of two taxa differentially expressed in oral microbiome samples, showing a similar trend of downregulation in individuals with MCI/AD [53]. More generally, *Lautropia mirabilis* was also identified as predominant in healthy subjects compared to periodontitis subjects [54,55], while *Prevotella* and *Veillonella* species were more abundant in caries-active subjects [55]. In our data, similarly, *Lautropia mirabilis* was significantly over-expressed in Control samples compared to AD (log2FC = −4.3, *p*-value < 0.001, Supplementary Table S7), and *Prevotella* and *Veillonella atypica* species were over-expressed in MCI/AD, which could, however, be a more general indication of decreased general dental health in MCI/AD subjects. In another study, *Veillonella parvula* significantly increased in the oral microbiome in AD [56]; however, we did not observe this difference in our study.

The employment of high-dimensional multi-omics data has provided exceptional resources for predictive research [57]. While substantial progress is still needed for omics-based diagnostics to become commonplace in clinical settings, this study illustrates the superior predictive power of integrating various omics datasets over single-source data. Identifying highly predictive molecular signatures enhances our comprehension of crucial molecular mechanisms underlying disease progression. A recent larger study of 455 patients demonstrated that individual datasets for DNA methylation, RNA, SNPs and proteomics yielded prediction with an accuracy of 0.63, 0.61, 0.59, and 0.58, respectively. However, after the integration of the four datasets, predictions were improved with a resulting accuracy of 0.95 (95% CI = [0.89–0.98]) [58], demonstrating that combining data from multiple platforms is a robust method for investigating biological systems. For our current, smaller study (n = 80), individual techniques provided sufficiently high accuracy on their own; however, they provide the option to combine multiple biomarkers in later studies if needed.

While omics studies of Alzheimer’s disease in saliva are still relatively rare, we can take advantage of the large body of proteomics studies carried out on brain tissue and CSF fluids to examine any overlap. To this end, we used a meta-analysis of Alzheimer’s brain proteomics datasets with deep coverage [59] and the web tool that has been made available to search against this resource [60]. The meta-analysis aggregates proteomic data from seven datasets [61,62,63,64], and we used it to query against proteins from our saliva protein clusters of interest, looking to highlight proteins found consistently differentially expressed in AD brains versus controls in brain and CSF proteomics sets. We found that three of the saliva proteins in the blue cluster of proteins discussed in this work (trend increasing with disease progression) were identified as up-regulated in nearly all brain AD proteomics datasets present in the meta-analysis (6 or more out of the 7 surveyed): secreted frizzled-related protein (up-regulated in saliva AD and MCI, and also present in our short list of saliva biomarkers, known to be elevated in human AD brains and mouse AD models [65]), Annexin A1 (ANXA1, up-regulated in saliva AD and MCI, anti-inflammatory with known role in reducing amyloid-β levels [66]), and Coronin 1A (up-regulated in saliva MCI vs. control). Similarly, molecular chaperone HSPB1 (AUC 0.8) from our short list of top five protein biomarkers is up-regulated in all the brain and CSF proteomics datasets above and was found to be down-regulated in saliva in the present study. HSPB1 is associated with senile plaques in AD and prevents the toxic effects of Aβ aggregates in vitro [67], and was found to be a key molecule in a regulatory network analysis integrating AD, Parkinson’s and MS [68]. Thus, taken together, the approaches provided in this study contribute various novel saliva biomarkers with high classification potential, several of which consistently appear in brain or CSF AD proteomics studies.

The connection and, hence, the interplay of protein abundances between the various body fluids is fascinating and complex, and comparisons of the various protein abundances have been studied via high-resolution mass spectrometry, including in plasma, saliva and CSF [69], highlighting that shared proteins are likely to be important in clinical applications, though the interplay and correlations between fluids, including plasma and saliva are likely to be complicated [70]. From observations in this current study, it is intriguing that highly ranked metabolites predominantly increase (accumulate in saliva) with disease progression. In contrast, promising protein biomarkers and those that correlate more highly with plasma pTau181 predominantly have levels that decrease in saliva with disease progression, and similarly for most of the microbiome species.

This study presents several limitations that should be taken into account. Firstly, the sample size is relatively small (*n* = 80), with only a subset of the cohort having microbiome data available. The study’s cross-sectional design means it can only capture associations at a single time point, which limits the ability to draw conclusions about disease progression. Several AD patients had initiated acetylcholine esterase inhibitor treatment at the time of sampling or just prior; therefore, given that timing, it is unlikely that acetylcholinesterase inhibitors affect the saliva metabolites or proteins as measured in this study. Indeed, there is no current evidence to support that even long-term acetylcholinesterase inhibitor treatment affects such metabolites in AD patients, and additionally, several of the top metabolite markers identified were either directly already known to be associated with AD (L-tyrosine) or involved in oxidative damage of proteins (3-chlorotyrosine) which is itself a marker of AD [37]. However, we cannot rule out that some saliva metabolites may have been altered by the acetylcholine esterase inhibitor treatment; thus, further studies will be needed to validate the findings in this study using untreated cohorts.

## 5. Conclusions

Collectively, these data highlight the potential of saliva as a powerful medium for early AD diagnosis, offering a practical solution for large-scale screening and monitoring. This pilot study provides several promising protein and metabolite candidate biomarkers for the detection and possibly progression of AD in saliva; correlations with plasma pTau181 levels provide additional support, as does placing them in the biological context of correlation networks. The practical utility and ease of access to saliva would make such markers compelling if validated in larger studies. In terms of study limitations, while the study incorporates various omics datasets, including proteomics, metabolomics, and microbiome data, larger and more longitudinal studies are needed to validate the candidate biomarkers and pathways identified. Saliva as a biofluid, while advantageous for being non-invasive, may have variable protein concentrations depending on external factors such as hydration and oral health.

## Figures and Tables

**Table 1:** Demographic information and various blood measurements in the format of mean (standard deviation) and ANOVA *p*-value.

Variable	CON (*n* = 40)	MCI (*n* = 20)	AD (*n* = 20)	*p*-Value
Sex: Male	21 (52.5%)	9 (45%)	12 (60%)	0.637
Age	74.9 (7.2)	77.8 (8.1)	78.0 (6.5)	0.182
MMSE	28.6 (1.6)	26.6 (3.1)	21.1 (3.3)	<0.001
APOE4: positive	6 (15%)	11 (55%)	10 (50%)	0.002
pTau181 (pg/mL)	3.2 (1.3)	4.8 (2.2)	6.6 (2.6)	<0.001
IL 6 (pg/mL)	19.1 (47.0)	16.9 (41.1)	14.4 (22.7)	0.915
TNF alpha (pg/mL)	1.6 (0.7)	1.9 (1.2)	2.1 (2.2)	0.33
Serum Vitamin B12 (pmol/L)	303.1 (91.3)	392.9 (186.0)	395.6 (120.5)	0.008
Active B12 Holotranscobalamin	61.8 (24.6)	83.6 (18.5)	63.0 (6.9)	0.192

Abbreviations: MMSE, Mini-mental state examination; APOE4 positive: APOE ε4 allele positive; IL 6: plasma interleukin 6; TNF alpha: plasma tumour necrosis factor alpha.

**Table 2:** Top 20 proteins and metabolites in order of their Pearson correlation with plasma pTau181.

Proteins	pTau181 Correlation	FDR-Adjusted *p*-Value	Metabolites	pTau181 Correlation	FDR-Adjusted *p*-Value
14-3-3 protein sigma	−0.49	0.004	3-Chlorotyrosine	0.46	0.001
Cornifin-A	−0.43	0.020	2-amino-2-methyl-1,3-propanediol	0.46	0.001
Small proline-rich protein 3	−0.42	0.020	L-Tyrosine	0.45	0.001
Aldehyde dehydrogenase, dimeric NADP-preferring	−0.42	0.020	Beta-gentiobiose	−0.44	0.001
Heme-binding protein 2	−0.41	0.020	Maltose	−0.44	0.001
14-3-3 protein eta	−0.41	0.020	Maltotriose	−0.44	0.001
Thioredoxin	−0.40	0.027	D-Malic acid	−0.44	0.001
Peroxiredoxin-6	−0.40	0.027	Urea	−0.43	0.001
Plasminogen activator inhibitor 2	−0.39	0.032	Cellotetraose	−0.43	0.001
Small proline-rich protein 2D	−0.37	0.046	Hydroxyphenyllactic acid	0.43	0.001
Protein LEG1 homolog	−0.37	0.046	Fumaric acid	−0.43	0.001
Heat shock-related 70 kDa protein 2	−0.37	0.046	Citraconic acid	−0.43	0.001
Carbonic anhydrase 1	0.37	0.046	Glycerol	−0.42	0.002
Annexin A3	0.36	0.046	Citramalic acid	0.42	0.002
Cystatin-S	−0.36	0.046	DL-3-aminoisobutyric acid	−0.39	0.004
Mesothelin	0.36	0.046	n-acetylneuraminic acid	0.39	0.005
Homeobox protein SIX5	−0.36	0.052	Rhamnose	0.38	0.006
Tubulin beta-6 chain	0.36	0.052	Cholesterol	0.37	0.009
Myosin-10	0.35	0.057	Leucyl-Leucine	−0.36	0.011
Tubulin beta-8 chain-like protein LOC260334	0.35	0.059	Beta-Alanine	−0.35	0.015

Ranking in increasing order of correlation *p*-value; the *p*-values presented were adjusted for multiple testing comparisons using the Benjamini and Hochberg FDR correction. Complete lists are available in Supplementary Tables S1 and S2.

**Table 3:** Functional characteristics of saliva protein and metabolite correlation modules showing significant correlation (*p*-value < 0.001) with MMSE scores: top-ranked pathways and top-ranked hub proteins.

Omics Platform	WGCNA Module	Top Ranked Pathways	Top 5 Hub Features
Proteins	Yellow (high positive correlation of module eigenprotein with MMSE, decrease with disease progression)	Vitamin B12 metabolism (SAA1, SERPINA3, transcobalamin-1) Folate metabolism (FOLR1, SAA1, SERPINA3)	Zinc-alpha-2-glycoprotein; Transcobalamin-1; Nucleobindin-1; Cystatin-C; Ribonuclease.T2
	Blue (high negative correlation of module eigenprotein with MMSE, increase with disease progression)	Pathogenic E-coli infection Parkin-ubiquitin proteasomal system pathway (various tubulins); S1P4/5/2 pathways; Purinergic signaling; T-cell activation; ALS (NEFH, NEFL, NEFM, PRPH, RAC1); Alzheimer’s disease (PSMA6 and various tubulins alpha and beta)	Various Histone2A and Histone2B proteins
Metabolites	Turquoise (high negative correlation of module eigenfeature with MMSE, increase with disease progression)	Arginine biosynthesis Valine, leucine and isoleucine biosynthesis Alanine, aspartate and glutamate metabolism	3-Chlorotyrosine; 2-amino-2-methyl-1,3-propanediol; L-Lysine; Glycylproline; Glycyl-Tyrosine
	Brown (high positive correlation of module eigenfeature with MMSE, decrease with disease progression)	Galactose metabolism Starch and sucrose metabolism Citrate cycle (TCA cycle)	Cellotetraose; Maltose; Maltotriose; Beta-gentiobiose; Beta-Alanine

Top-ranked pathways were determined using Cytoscape (for proteins, ranked in order of cosine similarity score) or MetaboAnalyst (for metabolites, top-ranked by enrichment *p*-values). Module members and kME values are listed in Supplemental Tables S3 and S4, and full tables are provided in Supplementary Information—section Pathway Analysis.

## Data Availability

The data presented in this study are available on request from the corresponding author. The data are not publicly available due to privacy and ethics considerations.

## Supplementary Materials

The following supporting information can be downloaded at https://www.mdpi.com/article/10.3390/metabo14120714/s1, Figure S1: MetaboAnalyst metabolite-disease interaction network for turquoise cluster of metab-olites increasing with disease progression (accessed on 29 August 2024); Figure S2: Volcano plot for microbiome AD vs Control using fold change and *p*-value from Table S7 (A), and WGCNA clusters for the microbiome analysis listed in detail in Table S6 (B), and module-trait correlation for microbiome analysis; Figure S3: Top progression biomarker performance, Transcobalamin 1. Table S1: Correlation of saliva proteins and blood plasma measures; Table S2: Correlation of saliva metabolites with and blood plasma measures; Table S3: WGCNA cluster membership and kME ranking for all saliva proteins; Table S4: WGCNA cluster membership and kME ranking for all saliva metabolites; Table S5: Phenotypic and plasma measurement characteristics of microbiome subcohort; Table S6: WGCNA cluster membership and kME ranking for all saliva microbiome species; Table S7: Differential expression statistics for microbiome data; Table S8: List of protein and metabolite biomarkers with ranking and AUC; Table S9: Differential expression details proteins; Table S10: Differential expression details metabolites.
