# Supplementary material for: Saliva Proteome, Metabolome and Microbiome Signatures for Detection of Alzheimer’s Disease
Source: Metabolites. 2024 Dec 19;14(12):714. doi: 10.3390/metabo14120714 (PMC11677859; doi:10.3390/metabo14120714)
Supplement: Supplementary file 1 [file metabolites-14-00714-s001.zip › metabolites-3228406 - Supplementary.pdf]

metabolites

MDPI

Supplementary Information

Saliva Proteome, Metabolome and Microbiome Signatures for Detection of Alzheimer's Disease

Maxime François [1], Dana Pascovici [2], Yanan Wang [3], Toan Vu [1], Jian-Wei Liu [4], David Beale [5], Maryam Hor [1], Jane Hecker [6], Jeff Faunt [7], John Maddison [8], Sally Johns [8] and Wayne Leifert [1,*]

[1] Nutrition and Health Program, Molecular Diagnostic Solutions Group, CSIRO Health & Biosecurity, Adelaide, SA 5000, Australia
[2] CSIRO Health & Biosecurity, Westmead, NSW 2145, Australia
[3] CSIRO Health & Biosecurity, Microbiomes for One Systems Health-Future Science Platform, Adelaide, SA 5000, Australia
[4] CSIRO Environment, Agricultural and Environmental Sciences Precinct, Acton, Canberra, ACT 2601, Australia
[5] Metabolomics Unit, CSIRO Environment, Ecosciences Precinct, Dutton Park, QLD 4001, Australia
[6] Department of Internal Medicine, Royal Adelaide Hospital, Adelaide, SA 5000, Australia
[7] Department of General Medicine, Royal Adelaide Hospital, Adelaide, SA 5000, Australia
[8] Aged Care Rehabilitation & Palliative Care, SA Health, Modbury Hospital, Modbury, SA 5092, Australia
* Correspondence: wayne.leifert@csiro.au

Supplementary Tables

Pathway analysis – Proteins

Pathway analysis was carried out using the online Cytoscape platform available at https://cytoscape.org/. Gene names corresponding to the proteins in each cluster of interest (blue and yellow protein cluster, available in the Supplementary tables) were submitted, and the pathways were ranked by cosine similarity. The output is shown below, including enrichment *p*-value (hypergeometric test). The top few pathways are summarised in Table 3 of the main manuscript.

Table S1. Top 10 pathways ranked by similarity; blue cluster of proteins increasing along disease severity (Control - MCI - AD).

Sort by Similarity p-Value Overlap

Pathway	Genes	Similarity	p-Value	Matched genes
WP2272 - Pathogenic Escherichia coli infection - Homo sapiens	16 / 56 unique genes	0.23	4.32e-14	ARPC2 ARPC4 ARPC5 CDC42 TUBA1A...
WP2359 - Parkin-ubiquitin proteasomal system pathway - Homo sapiens	12 / 73 unique genes	0.15	1.60e-7	TUBA1A TUBA1B TUBA1C TUBA3E TUBA4A...
S1P4 pathway (v2.0)	6 / 14 unique genes	0.12	3.48e-6	CDC42 GNA12 GNA13 GNAI1 GNAI2...
WP4900 - Purinergic signaling - Homo sapiens	7 / 33 unique genes	0.11	4.28e-5	GNAI1 GNAI2 GNAO1 GNAS GNAT1...
T cell activation	6 / 55 unique genes	0.11	3.37e-3	ACTR2 ACTR3 ARPC2 ARPC4 ARPC5...
S1P5 pathway (v2.0)	4 / 8 unique genes	0.10	1.58e-4	GNA12 GNAI1 GNAI2 GNAO1
WP2447 - Amyotrophic lateral sclerosis (ALS) - Homo sapiens	5 / 38 unique genes	0.10	3.90e-3	NEFH NEFL NEFM PRPH RAC1
WP5124 - Alzheimer's disease - Homo sapiens	13 / 263 unique genes	0.10	3.37e-3	PSMA6 TUBA1A TUBA1B TUBA1C TUBA3E...
WP2059 - Alzheimer's disease and miRNA effects - Homo sapiens	13 / 268 unique genes	0.10	3.54e-3	PSMA6 TUBA1A TUBA1B TUBA1C TUBA3E...
S1P2 pathway (v2.0)	6 / 26 unique genes	0.09	1.11e-4	GNA12 GNA13 GNAI1 GNAI2 GNAO1...

Table S2. Top 10 pathways ranked by similarity; yellow cluster of proteins decreasing along disease severity (Control - MCI - AD).

Sort by ◉ Similarity ○ p-Value ○ Overlap

Pathway	Genes	Similarity	p-Value	Overlapping genes
WP1533 - Vitamin B12 metabolism - Homo sapiens	3 / 55 unique genes	0.09	3.72e-1	SAA1 SERPINA3 TCN1
WP176 - Folate metabolism - Homo sapiens	3 / 70 unique genes	0.08	3.72e-1	FOLR1 SAA1 SERPINA3
WP3890 - Nanomaterial-induced inflammasome activation - Homo sapiens	1 / 8 unique genes	0.08	3.72e-1	CTSB
PID v2.0 Endogenous TLR signaling (v2.0)	3 / 26 unique genes	0.07	1.81e-1	CD14 RHOA SAA1
WP186 - Homologous recombination - Homo sapiens	1 / 13 unique genes	0.07	3.72e-1	RAD54B
PID v2.0 Glypican 3 network (v2.0)	1 / 7 unique genes	0.07	3.72e-1	FURIN
PID v2.0 p75(NTR)-mediated signaling (v2.0)	4 / 70 unique genes	0.07	1.81e-1	FURIN RHOA RHOB RHOC
PID v2.0 Syndecan-1-mediated signaling events (v2.0)	2 / 40 unique genes	0.07	3.72e-1	COL6A1 PPIB
WP4217 - Ebola virus pathway in host - Homo sapiens	5 / 139 unique genes	0.06	2.35e-1	CTSB FOLR1 RHOA RHOB RHOC
WP5065 - SARS-CoV-2 altering angiogenesis via NRP1 - Homo sapiens	1 / 7 unique genes	0.06	3.72e-1	FURIN

Pathway analysis – Metabolites

Pathway analysis was carried out using the MetaboAnalyst Pathway analysis tool (https://www.metaboanalyst.ca/) for the two clusters of interest for metabolites, turquoise and brown, using the compound names available in the supplementary tables. The default parameters were used: KEGG Homo Sapiens database, and enrichment via hypergeometric test. The top 10-ranked pathways are summarised in the table below, and the top three pathways are highlighted in Table 3 in the main manuscript.

Turquoise cluster compound names as input, top 10 pathways	Total	Expected	Hits	Raw p	LOG10(p)	Holm adjust	FDR	Impact
Arginine biosynthesis	14	0.26667	4	9.40E-05	4.0269	0.00752	0.00752	0.06091
Valine, leucine and isoleucine biosynthesis	8	0.15238	3	0.000328	3.4843	0.025903	0.013115	0
Alanine, aspartate and glutamate metabolism	28	0.53333	4	0.001596	2.797	0.12447	0.04112	0.38542
Phenylalanine, tyrosine and tryptophan biosynthesis	4	0.07619	2	0.002056	2.687	0.15831	0.04112	1
Phenylalanine metabolism	8	0.15238	2	0.009149	2.0386	0.69531	0.14638	0.35714
Arginine and proline metabolism	36	0.68571	3	0.029158	1.5352	1	0.34079	0.3686
Histidine metabolism	16	0.30476	2	0.035672	1.4477	1	0.34079	0.22131
Neomycin, kanamycin and gentamicin biosynthesis	2	0.038095	1	0.037744	1.4231	1	0.34079	0
Valine, leucine and isoleucine degradation	40	0.7619	3	0.038339	1.4164	1	0.34079	0.03348
Tyrosine metabolism	42	0.8	3	0.043426	1.3622	1	0.34741	0.16435

Brown cluster compound names as input, top 10 pathways	Total	Expected	Hits	Raw p	LOG10(p)	Holm adjust	FDR	Impact
Galactose metabolism	27	0.29143	7	3.28E-09	8.4843	2.62E-07	2.62E-07	0.54111
Starch and sucrose metabolism	18	0.19429	3	0.000772	3.1123	0.060994	0.02838	0.12786
Citrate cycle (TCA cycle)	20	0.21587	3	0.001064	2.9729	0.083013	0.02838	0.16653
Pyruvate metabolism	23	0.24825	3	0.00162	2.7904	0.12477	0.032408	0.19137
Alanine, aspartate and glutamate metabolism	28	0.30222	3	0.002899	2.5378	0.22029	0.046377	0.0024
Glycolysis / Gluconeogenesis	26	0.28063	2	0.030616	1.514	1	0.40822	0.09785
Glyoxylate and dicarboxylate metabolism	32	0.3454	2	0.044987	1.3469	1	0.51414	0.03175
Amino sugar and nucleotide sugar metabolism	42	0.45333	2	0.073327	1.1347	1	0.6518	0
Tyrosine metabolism	42	0.45333	2	0.073327	1.1347	1	0.6518	0.02463
Ascorbate and aldarate metabolism	9	0.097143	1	0.09328	1.0302	1	0.74624	0

Plasma pTau181differential expression statistics by Condition and APOE4 status.

Tukey Honest Significant Differences (Tukey HSD) generated for Condition, APOE4 status, and their interaction; the pTau181 levels are significantly different between all three conditions. Adjusted p-values < 0.05 are highlighted in gray.

Comparison	Mean difference	Lower end point	Upper end point	Adjusted p-value
AD-CON	3.373552	2.083396	4.663709	7.21E-08
AD-MCI	1.813227	0.329808	3.296645	0.01262
MCI-CON	1.560326	0.270169	2.850482	0.01375
APOE4+-APOE4-	0.164948	-0.76201	1.091906	0.723879
AD:APOE4+-CON:APOE4-	3.850367	1.778841	5.921893	9.87E-06
AD:APOE4--CON:APOE4-	2.892767	0.821241	4.964293	0.001501
AD:APOE4+-CON:APOE4+	3.863272	0.899823	6.826721	0.003703
CON:APOE4+-AD:APOE4-	-2.90567	-5.86912	0.057777	0.057826
AD:APOE4+-MCI:APOE4+	2.454845	-0.05257	4.962261	0.058457
MCI:APOE4--CON:APOE4-	1.75734	-0.4007	3.91538	0.175745
AD:APOE4+-MCI:APOE4-	2.093026	-0.54372	4.729775	0.198195
MCI:APOE4+-CON:APOE4-	1.395522	-0.60244	3.393479	0.328083
MCI:APOE4+-AD:APOE4-	-1.49725	-4.00466	1.010171	0.50551
CON:APOE4+-MCI:APOE4-	-1.77025	-4.7948	1.254312	0.527831
MCI:APOE4+-CON:APOE4+	1.408427	-1.50407	4.320925	0.71769
AD:APOE4--MCI:APOE4-	1.135427	-1.50132	3.772175	0.805256
AD:APOE4+-AD:APOE4-	0.957599	-1.60882	3.524022	0.883025
MCI:APOE4+-MCI:APOE4-	-0.36182	-2.94117	2.217533	0.998445
CON:APOE4+-CON:APOE4-	-0.01291	-2.55981	2.534	1

WGCNA Analysis Details

The steps undertaken for WGCNA analysis are as summarized in section 3.8 of our book chapter **Application of WGCNA and PloGO2 in the Analysis of Complex Proteomic Data, https://link.springer.com/protocol/10.1007/978-1-0716-1967-4_17**

Briefly, they consist of:

- Calculating the soft threshold
- Calculating the adjacency matrix
- Building the hierarchical clustering using the TOM dissimilarity measure followed by dynamic tree cut and merging similar clusters
- Calculating module eigenproteins
- Calculating kMEs and hub proteins

Some of the example intermediary outputs are shown below for the Saliva Proteomic dataset.

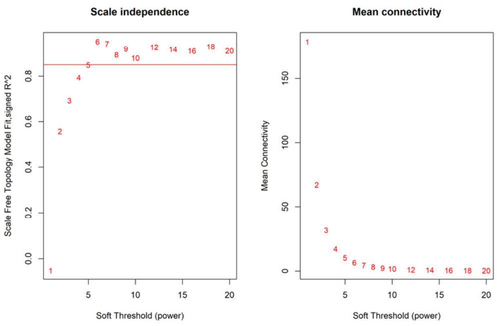

Soft threshold for saliva proteomics

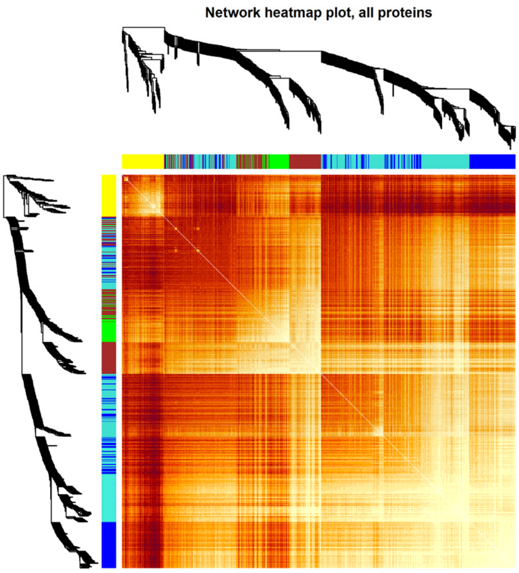

Correlation matrix for saliva proteomics

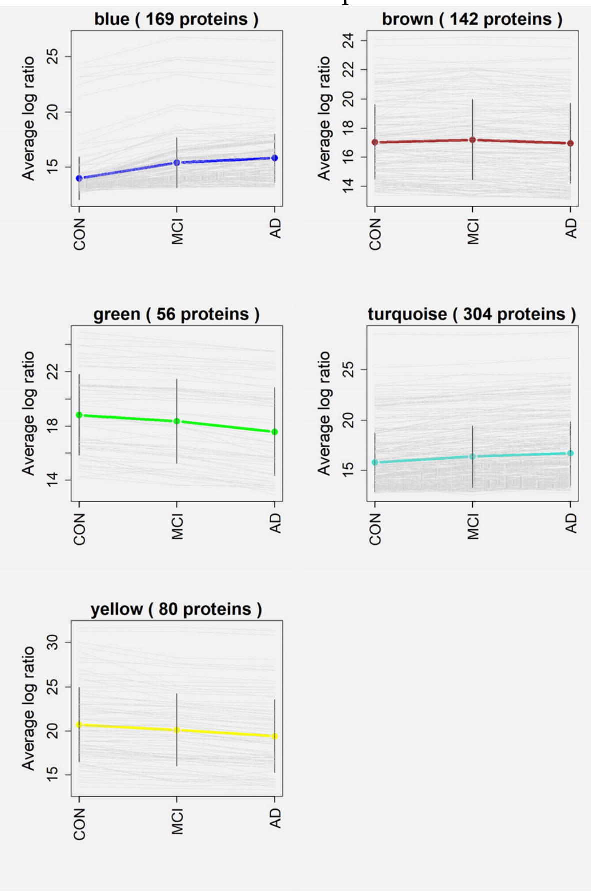

Overlayed patterns of proteins in the clusters for saliva proteomics

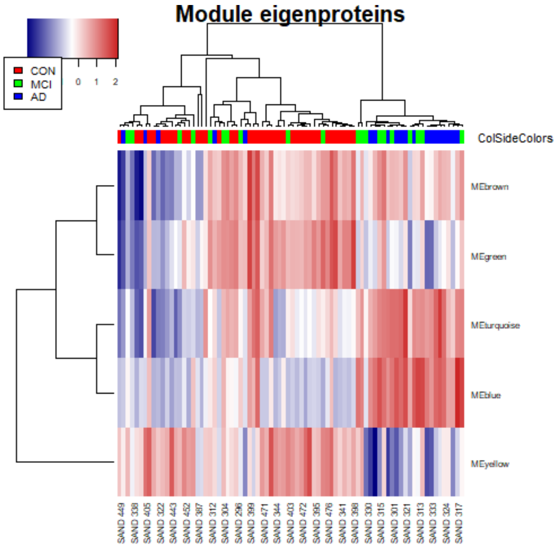

Clustering of resulting module eigenproteins—saliva proteomics.

Supplementary Figures

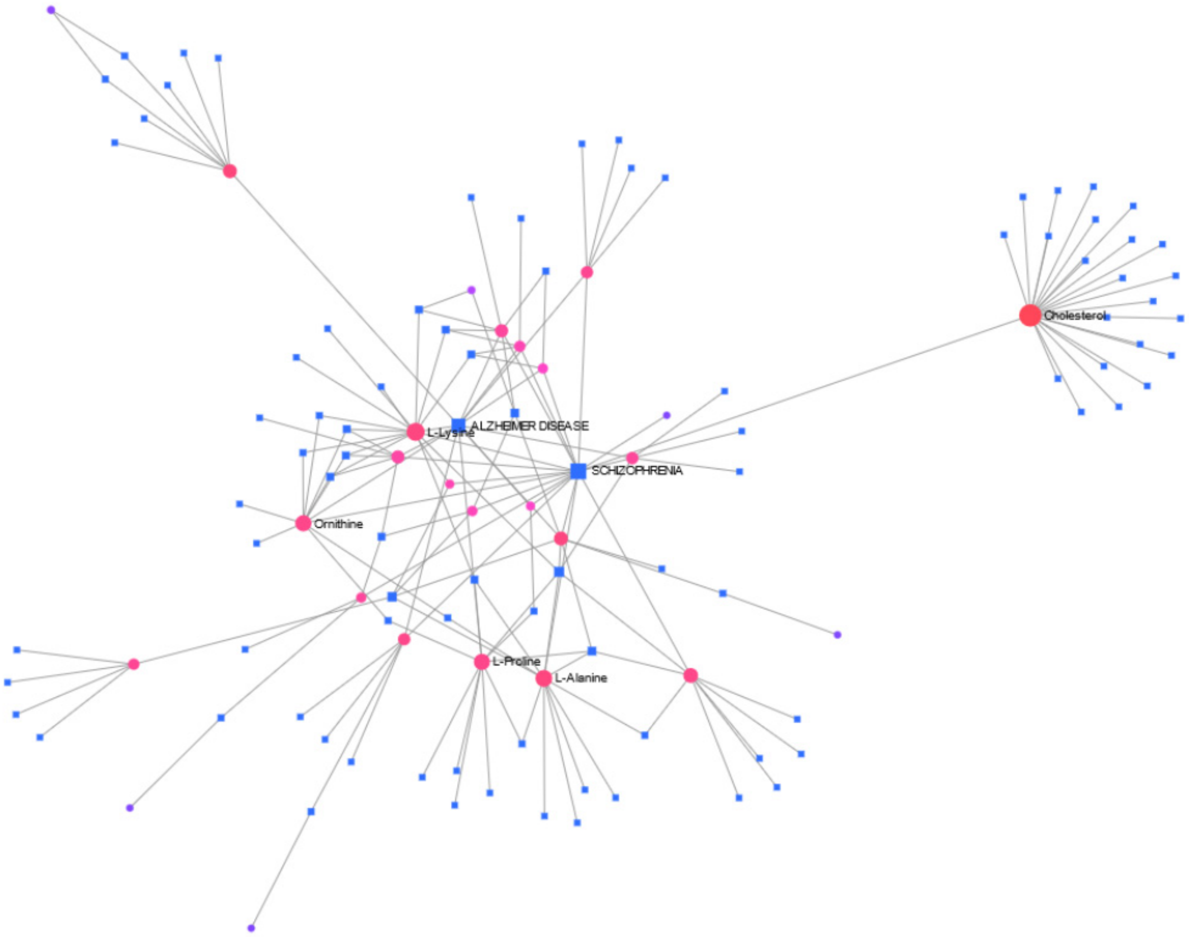

Figure S1. MetaboAnalyst metabolite-disease interaction network for turquoise cluster of metabolites increasing with disease progression (accessed on 29 August 2024). Input metabolites can be found in Supplementary Table 4, filtering for "turquoise" module membership. MetaboAnalyst "*Network Analysis*" tool was selected using default parameters; the image below represents the first subnetwork generated by the analysis.

Metabolites from network connected to ALZHEIMER DEGREE node include: L-Tyrosine, L-Phenylalanine, L-Proline, L-Threonine, L-Histidine, L-Lysine, L-Cystine, Ornithine, L-Glutamine, L-Valine, L-Tryptophan.

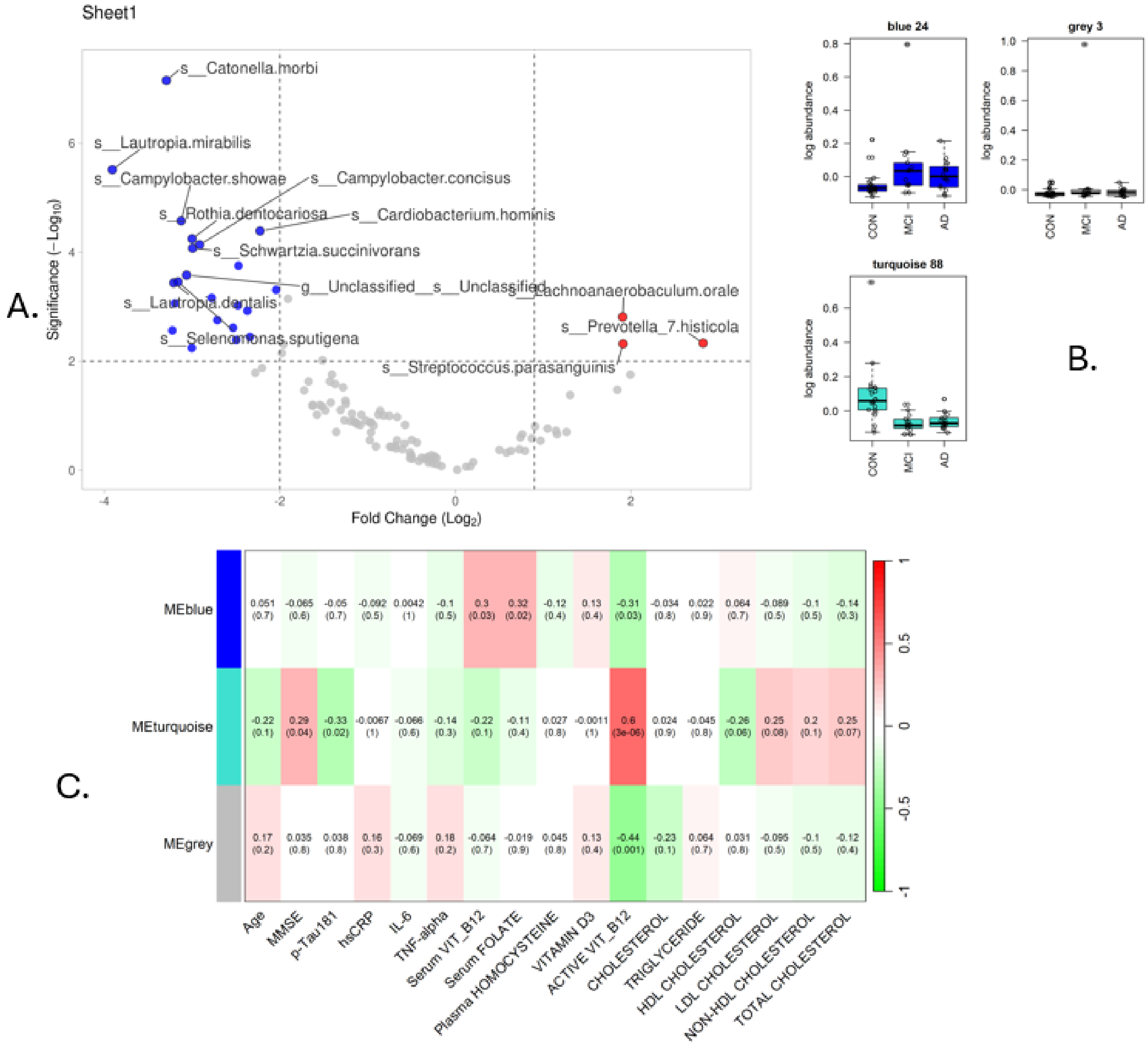

Figure S2. Volcano plot for microbiome AD vs Control using fold change and p-value from Supplementary Table 7 (A.), and WGCNA clusters for the microbiome analysis listed in detail in Supplementary Table 6 (B.), and module-trait correlation for microbiome analysis.

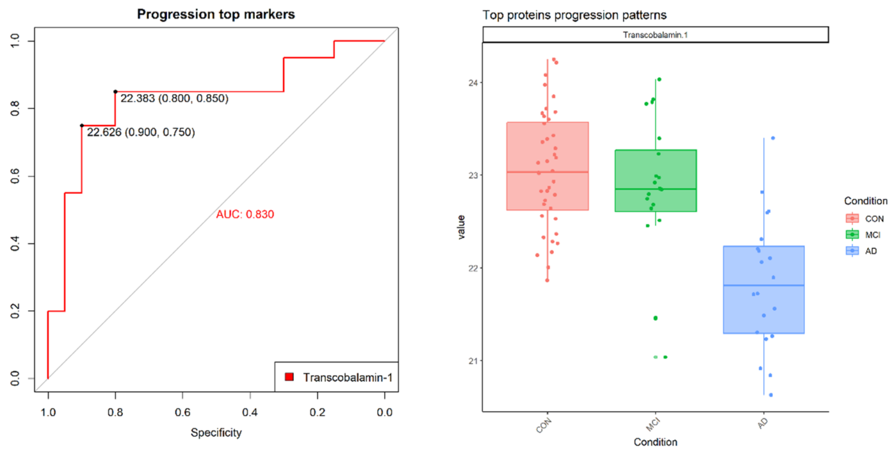

Figure S3. Top progression biomarker performance, Transcobalamin 1. Performance (ROC, AUC) is reported using the MCI and AD samples only (n = 20 each). Boxplot shows values for all available samples (n = 80).
